# Hyperledger Fabric Access Control System for Internet of Things Layer in Blockchain-Based Applications

**DOI:** 10.3390/e23081054

**Published:** 2021-08-16

**Authors:** Adnan Iftekhar, Xiaohui Cui, Qi Tao, Chengliang Zheng

**Affiliations:** Key Laboratory of Aerospace Information Security and Trusted Computing, Ministry of Education, School of Cyber Science and Engineering, Wuhan University, Wuhan 430072, China; adnan@whu.edu.cn (A.I.); qitao17@whu.edu.cn (Q.T.); chengliang@whu.edu.cn (C.Z.)

**Keywords:** Hyperledger Fabric, Internet of Things, IoT, access control system, blockchain

## Abstract

Blockchain-based applications are gaining traction in various application fields, including supply chain management, health care, and finance. The Internet of Things (IoT) is a critical component of these applications since it allows for data collection from the environment. In this work, we integrate the Hyperledger Fabric blockchain and IoT devices to demonstrate the access control and establish the root of trust for IoT devices. The Hyperledger Fabric is designed to be secure against unwanted access and use through encryption protocols, access restrictions, and cryptography algorithms. An attribute-based access control (ABAC) mechanism was created using Hyperledger Fabric components only to gain access to the IoT device. Single board computers based on the ARM architecture are becoming increasingly powerful and popular in automation applications. In this study, the Raspberry Pi 4 Model B based on ARM64 architecture is used as the IoT device. Because the ARM64 architecture is not supported by default, we build executable binaries and Docker images for the ARM64 architecture, using the Hyperledger Fabric source code. On an IoT device, we run the fabric node in native mode to evaluate the executable binaries generated for the ARM64 architecture. Through effective chaincode execution and testing, we successfully assess the Hyperledger fabric blockchain implementation and access control mechanism on the ARM64 architecture.

## 1. Introduction

The Internet of Things (IoT) is a critical component of industrial automation [1]. IoT is a broad term that refers to many smart sensors and micro-controllers that collect data from their surroundings. These sensors serve as the foundation for contemporary technologies, such as smart homes, smart cities, smart grids, smart health systems, and wearable gadgets [2]. Despite its enormous potential, numerous use cases, rapid expansion, and several futuristic visions, IoT still faces several obstacles. Some significant obstacles are data privacy, security, and centralization of IoT devices [3]. The majority of IoT solutions are centralized and rely on cloud computing for data storage and other services.

In comparison to a centralized approach, the blockchain is a decentralized and distributed ledger. It is a tamper-proof data sharing and decentralized network governance system [4]. The IoT sector is rapidly adopting blockchain technology because of its provenance in security and traceability [5]. Existing security concerns in the IoT can be solved through the usage of the decentralized architecture of blockchain technology, which is built with security and privacy as built-in components [6]. Blockchain encryption prevents anyone from overwriting existing data records. Using blockchain to store IoT data provides another layer of security to avoid unwanted attacks. IoT devices are vulnerable to DDoS assaults, malicious attacks, and data breaches. Protecting data throughout the IoT ecosystem is a major problem for businesses. The combination of IoT with blockchain enables safe machine-to-machine transactions while reducing inefficiencies and improving security. Customers of IoT require data and insights from IoT devices promptly, affordably, and reliably. The blockchain can be the central ledger for all of this. One of the major obstacles to combining blockchain with IoT is the short battery life of some IoT devices. Some IoT gadgets are always linked to electricity and Wi-Fi, so there are no actual limitations, while many IoT gadgets are not. Moreover, a computation- and bandwidth-intensive blockchain transaction system cannot run on a little device. So it may need to employ a rely on a gateway or equivalent device, such as Raspberry Pi single board computers. So these ecosystems will have to be cooperative by nature.

Blockchain is a notion that was developed out of encryption algorithms, technology, and new concepts for the exchange of economic value in a decentralized manner [7]. Transferring information between parties on the blockchain is accomplished by using digital signatures and the consensus mechanism [8]. Compared to blockchain technology, distributed ledger technology (DLT) solely depends on digital signatures to ensure the ledger’s integrity. Bitcoin is an example of peer-to-peer financial exchange, as well as being the world’s first practical implementation of blockchain technology [9]. Smart contracts are software programs used to perform business logic in blockchain networks [10]. One of the most popular recent developments in using smart contracts with blockchain technology is the traceability of the assets in the supply chain management [11].

Blockchain can make end-to-end tracking in the supply chain more visible and accurate [12]. Organizations can digitize their physical assets using blockchain technology to establish a decentralized and immutable record of transactions [13]. It will make it easy to monitor assets from manufacture to delivery or usage by the end-user [14]. Blockchain technology is also proven to be the optimal tool for driving the food sector forward [15]. Current applications of distributed ledger technology in the food sector include tracking food supply chains and managing food safety procedures [16]. Internet of Things devices that use GPS and other technologies to track and authenticate items and shipments in the supply chain is an efficient method to streamline the process [17]. It also allows them to keep an eye on the storage conditions of items, which helps to improve quality control throughout the whole supply chain [18].

In order to cooperate in centralized systems, different parties have to trust each other or hire a third party. It hinders the interoperability of various IoT apps and services [19]. On the other hand, decentralization would offer many benefits compared to centralized infrastructure if accomplished. The most important results of IoT decentralization is the distributed consensus amongst IoT devices [20]. If it is correctly regulated, it can enhance the security of IoT systems and provide greater privacy for consumers, using efficient data protection methods. Decentralized Internet of Things solutions can handle a large volume of transactions and scale to many peers to achieve consensus without the intervention of a trusted central authority. Most notably, the development of the blockchain has provided a means of overcoming distributed consensus constraints in a decentralized environment for large-scale applications [21]. It is increasing the use of blockchain technology in the development of decentralized Internet of Things solutions.

**Figure 1:**
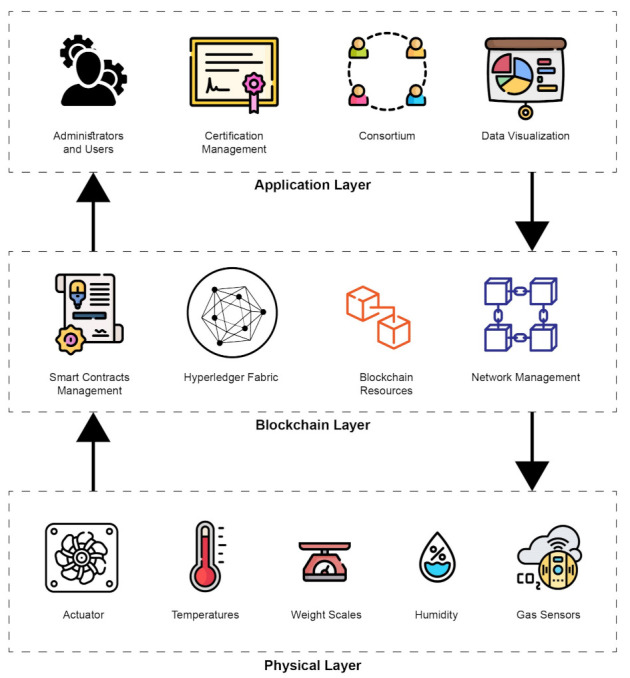
An overview of the blockchain-based supply chain management system architecture [16].

Hyperledger fabric is an enterprise-ready open-source blockchain development platform [22]. In addition to exhibiting blockchain characteristics, such as a decentralized ledger, tamper-proof data sharing also offers a more efficient consensus mechanism with higher throughput, using RAFT Orderer Service [23]. In this work, we used the Hyperledger Fabric blockchain technology built-in features to demonstrate an access control system that offers dynamic access control management for IoT devices in the blockchain network. We implemented a general system design as shown in Figure 1. We created an experimental network to demonstrate a real-world IoT-blockchain integration scenario. In our experimental network, we have three member organizations, Org1, Org2, IoT, and one Orderer organization with a solo orderering service.

The rest of this paper is structured as follows. Section 2 introduces the background work. Section 3 introduces the Hyperledger Fabric and its core components. The Fabric access control mechanism, Fabric policies and resource access control lists are discussed in Section 4. The architecture design and implementation of our system is demonstrated in Section 5 followed by the conclusion in Section 7, performance limitations in Section 6 and future work in Section 8.

## 2. Background Studies

This section provides an overview on the related work in the areas of the Internet of Things and blockchain technology.

**Figure 2:**
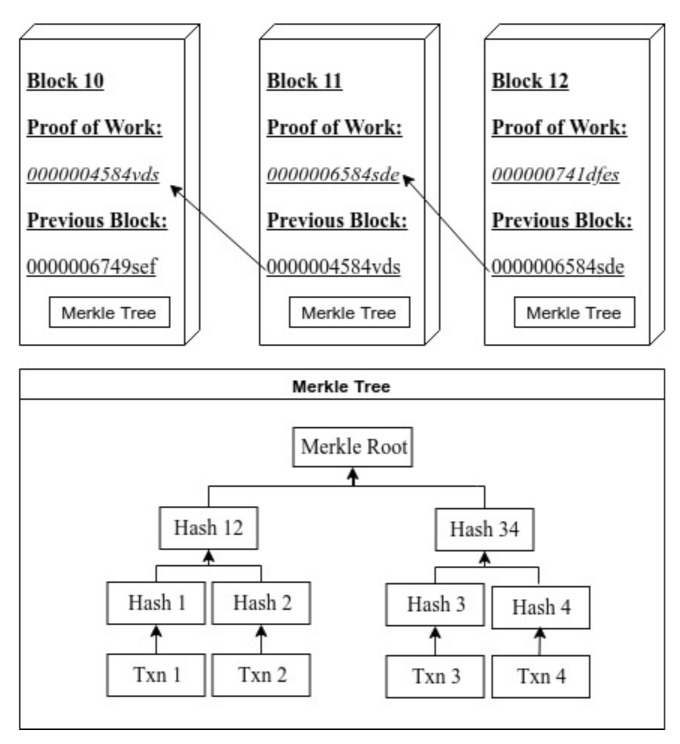
Blocks forming the blockchain using hash signature [15].

Bitcoin evolved from the first blockchain-based application in the world [24]. It is a link-list type data set of transactions that is accessible by a large number of parties. Digital signatures and cryptographic hash algorithms protect its integrity [25]. This list is disseminated across multiple computers through peer-to-peer networks in near real time. It is almost difficult for any changes to be introduced into prior transactions, and it is straightforward to identify any unlawful changes in data [11]. Blockchain transactions are stored in blocks that are arranged sequentially. It is illustrated in Figure 2, where each block contains several transactions linked with the previous block by a hash signature.

Each block has a block header in which a unique Merkle Root Tree identifies the current block transactions hash value [26]. A genesis block is an initial block on a blockchain, and all subsequent blocks are linked to it. A blockchain can be divided into three categories as summarized in the Table 1.

The security of the blockchain derives from the innovative use of cryptography, a consensus algorithm, and peer-to-peer networking. When someone joins the blockchain network, it is a must to import all of the blockchain transactions. The system is capable of ascertaining whether everything is in order. When new blocks are generated, they are distributed to other nodes through the network. Each node then validates the authenticity of the block. Each node adds the information to its blockchain if it is legitimate. As a result, the network’s nodes attain a consensus. They concur as to which blocks are legitimate and which are invalid. The network’s other nodes reject blocks that have been tampered with. To tamper with the public blockchain, we must compute all proofs of work and gain greater than 50% control of the peer-to-peer network, which is nearly impossible. A thorough examination of a blockchain with its different elements, architecture, and functioning methods can be reviewed from the article of Singhal et al., “How Blockchain Works?” [27].

The present methods for access control are not intended for restricted devices. They are mostly centralized, bringing about scalability and interoperability, ending security problems that need users to share their data with third parties [28]. Adopting a decentralized access control strategy offers many advantages. However, it also has disadvantages, such as difficulties updating access control policies. However, updates may be quickly made through smart contracts [29].

Putra et al. proposed a blockchain-based trust and reputation system (TRS) for IoT access control, which gradually evaluates and calculates each participating node’s trust and reputation score to generate a self-adaptive, trustworthy access control system. Trust and reputation are explicitly incorporated in the attribute-based access control policy, allowing different nodes to be allocated to different access right levels, resulting in dynamic access control policies [30].

Liu et al. proposed a capability-based IoT access control architecture leveraging blockchain and decentralized device identification and access control identifiers. They provided a technique to create a systematic view of system interactions, improving safety. They constructed a prototype proof of the recommended approach and evaluated the prototype, using real-world situations [31].

Pinno et al. proposed ControlChain, which is a blockchain-based access control authorization framework. They show ControlChain’s feasibility using the E-ControlChain, a proof-of-concept designed to run on the Ethereum network. Finally, the authors conducted a study of E-ControlChain’s cost and performance, utilizing Raspberry Pi as an IoT device [32].

Patel et al. provided an overview of the available blockchain-based security approaches for Internet of Things access control in vehicular ad hoc networks and healthcare; the supply chain is presented in this review article [33].

Zhang et al. proposed a smart contract-based architecture consisting of many Access Control Contracts (ACCs), one Judge Contract (JC), and one Register Contract (RC) to perform distributed, trustworthy IoT access control systems. Each ACC offers one access control mechanism for a subject-object pair and, by evaluating the behavior of the subject, implements both predetermined access right validation and dynamic access right validation [34].

Ouaddah et al. demonstrated that blockchain might be a desirable solution for dealing with IoT access control issues. They proposed FairAccess as a novel decentralized pseudonymous and privacy-preserving authorization management system that makes use of the consistency of the distributed ledger technology (blockchain) to handle access control on behalf of restricted devices [35].

Yang et al. presented a new dual-access control mechanism that is self-adaptive for both regular and emergency scenarios in the health care system. Using a password-based break-glass access method, healthcare personnel with sufficient attribute secret keys can have data access privileges in regular applications; in emergency applications, patient’s past medical data can be retrieved [36].

Pinno et al. proposed a blockchain-based IoT authorization framework. The architecture is user-friendly, completely decentralized, scalable, fault-tolerant, and compatible with a wide range of today’s IoT access control models. The design also includes a safety mechanism to build relationships between people, devices, and a group of both, allowing characteristics for these relationships to be assigned and used in the access control authorization [37].

## 3. Hyperledger Fabric

This section overviews the Hyperledger Fabric and its components, design, reference architecture, and overall enterprise readiness. Hyperledger Fabric is an open-source technological blockchain framework. Open source, open standards, and open architecture belong to the open-source initiative [38]. An open-source effort enables users to integrate and tailor the system to meet their own needs. It benefits consumers by helping them avoid vendor lock-in. Businesses are frequently obliged to comply with a variety of industry compliance and technology governance standards. Hyperledger Fabric blockchain technology is an enterprise-ready piece of open-source software that powers a corporate network. It also assists in resolving challenges related to compliance and technology governance, which may have a compounding effect on the cost of technology consumption, governance, and maintenance. It is a permissioned blockchain platform that is suitable for corporate use. It is developed in golang and communicates with the rest of the system using the gRPC communication mechanism. The Hyperledger Software Development Kits (SDKs) are available in Java, Node.js, and golang. The official documentation of Hyperledger Fabric is available online [23].

### 3.1. Hyperledger Fabric Components

Hyperledger Fabric is a blockchain implementation intended for use in deploying a modular and extensible architecture design. It allows for alternative implementations to be plugged in and implemented with a modular subsystem architecture as the system grows in complexity. The three critical components of the design are the membership service provider (MSP), dedicated orderer service, and peer nodes.

#### 3.1.1. Membership Service Provider (MSP)

The Hyperledger Fabric Certification Authority is a dedicated X509-based identification service to issue identities in the network. Besides Fabric CA, any other service that provides X509-based PKI infrastructure can be used to issue identity certificates.

#### 3.1.2. Dedicated Ordering Service

The Orderer serves as the network’s communication backbone. The Orderer is responsible for ensuring that the ledger state is consistent across the network. Consensus is established in Fabric via the Orderer, and the Orderer is accountable for maintaining the transaction’s order. The Fabric provides a solo orderer service for experimental purposes, and for production purposes, it provides a RAFT-based ordering service [39].

### 3.2. Peer Nodes

Smart contracts and the maintenance of the ledger are the responsibility of peers. The Fabric has two particular types of peer nodes: “anchor peers” and “endorser peers”. Anchor peers are accessible outside of the organization. The anchor peers receive the network’s data blocks and distribute them to the other peers. An organization creates a cluster of anchor peers to avoid a single point of failure. Peers may be designated as endorsers or assume the position of an endorser peer. Clients send an invocation request for the smart contract (chaincode) to the endorser peers. Upon receipt of the invocation request, the endorser peers simulate and validate the chaincode transaction.

### 3.3. Permissioned Network

In a public blockchain network, people download the software and immediately begin transacting anonymously. It is not an acceptable method of operation in business networks. In enterprise networks, anonymity is not acceptable. Members of business networks are always known by their identifiers and allocated responsibilities. The Hyperledger Fabric is a permission-based network that allocates transactions to recognized identities and responsibilities. All users and components on the Hyperledger Fabric network must be authenticated. The Hyperledger Fabric assigns these entities their network identities via Membership Service Providers (MSP) and Certification Authorities (CA), which employ a Public Key Infrastructure (PKI) to approve and validate users and components.

### 3.4. Confidential Transactions

Confidentiality from unrelated parties is a critical characteristic in many business settings. Occasionally, business networks choose to keep their transactions extremely secret from unrelated parties and disclose them exclusively to the counter party. Hyperledger Fabric provides channel capability that enables transactions to be private between specified parties. Each channel has its ledger, and several channels may exist among consortium members connected to the same network.

### 3.5. Hyperledger Fabric Policies

The consortium’s members create several policies, decisions, rules, and regulations that govern the consortium’s operation. Typically, the consortium makes decisions that are decentralized. Numerous administrators from member organizations vote by majority to make changes to the network that impact the consortium or business network’s members. A decentralized decision-making system of this nature requires governance and decision-making frameworks. By utilizing rules, the Hyperledger Fabric technology enables decentralized administration.

### 3.6. Application Development and Integration

**Figure 3:**
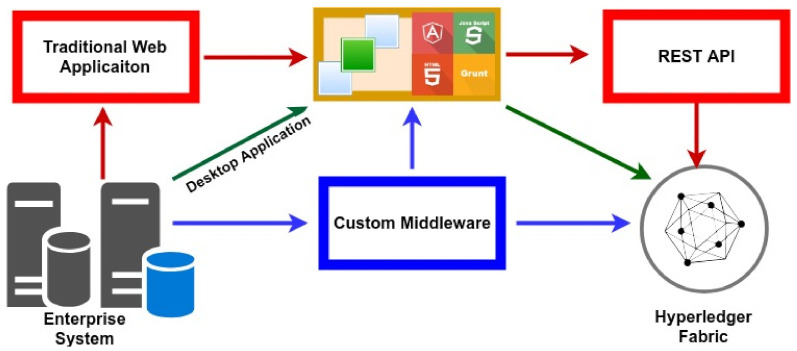
Application development and integration [16].

Integrating the Hyperledger Fabric blockchain with an existing corporate system is quite straightforward. Each company in the network may customize the interaction system to meet its requirements. Fabric front-end apps can be created independently utilizing RESTful APIs as middle-ware or using one of the Hyperledger Fabric SDKs; see Figure 3.

### 3.7. Fabric Certification Authority

The actors in the Fabric blockchain network can be categorized into human actors, such as admins, users, and machine actors, such as Orderers, Peers, and Applications. All actors and components need an identity to participate in the blockchain network in an x509 certificate. These certificates contain information about the holder. These certificates also hold some additional attributes and roles to determine the privileges in the network. The Hyperledger Fabric provides a built-in certification authority (CA) to issues the identities into the network [40]. Other than the Fabric CA, any certification authority that issues x509 certificates can be used.

**Figure 4:**
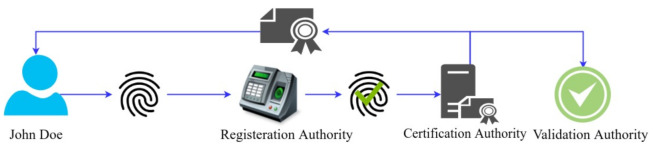
X509 certificate issuance steps.

The procedure of the x509 certificate issuance is shown in Figure 4. It is a two-step process. In the first step, the certification authority (registrar) creates the identity into the system and provides the authority holder’s credentials. In the second step, the identity holder enrolls the identity to obtain its x509 certificate. In a non-human actor, the respective admin of the network enrolls and initializes the component.

## 4. Access Control in Hyperledger Fabric

Access control is a critical characteristic of a secure, permissioned blockchain. Typically, the access control method for accessing the chaincode by the member organizations is embedded in the chaincode itself and is enforced during transaction processing on several endorsing peers. The result is validated by transaction consensus. Additional access control techniques can be integrated into the contract-interacting application levels.

On the basic level, the access control system originates from the consortium level planning. The consortium decides the Hyperledger Fabric policies and rules, which become part of the genesis block and governs the whole system.

### 4.1. Hyperledger Fabric Policies

The Hyperledger Fabric policies define the rules that must be followed while accessing or updating the network and channel settings. The consortium defines the initial set of policies, as it is responsible for providing a fine-grained access control foundation over the various components of the blockchain network. These policies are encoded into the configtx.yaml file and become part of the genesis block. These policies define which elements or configurations can be changed and by whom as well as how these changes will be implemented. For example, any admin from the member organization can add an anchor peer for its organization. To change any channel configurations, most admins from the member organizations must agree. The members provide their agreements by signing the channel configuration transaction. The MSP plays an essential part in the implementation of the access control system. The Certification Authority (CA) embedded requires attributes for the x509 certificate.

The policies are of two types: the signature policy and the Implicit Meta policy. The Implicit Meta policies refer to the other policies. The policy definition has two parts to the policy type (“Signature” OR “Implicit Meta”) and the Rule. The signature policies are applicable at all levels, while the Implicit Meta policies are applicable at channel configuration only.

### 4.2. Policies Configuration and Implementation

The policies in the fabric network are defined in the way of a hierarchy. Each policy has its own dedicated section. The top-level hierarchy in policies is “/Channel”. It is exceptionally restrictive because any change in the channel level policy affect the whole network. The next level is “/Channel/Orderer”, “/Channel/Application”, and “/Channel/Application/<Organization>”.

**Figure 5:**
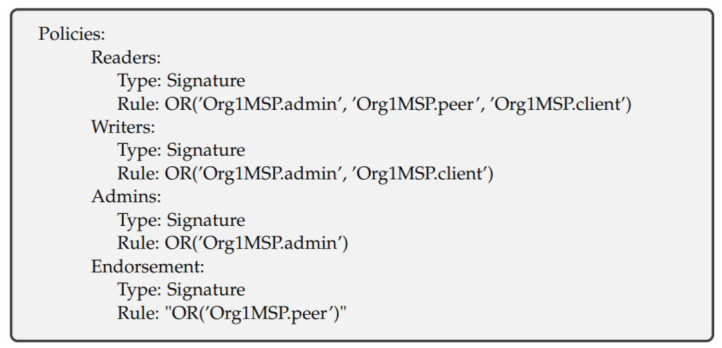
Member organizations’ policies, except the Orderer organization.

**Figure 6:**
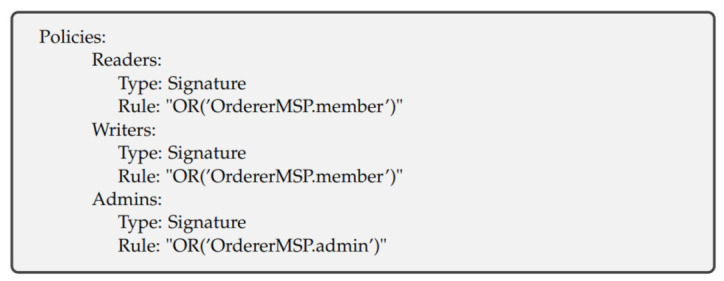
Orderer organization policy in experimental network.

The policies have such names as “/Channel/Application/<Organization>/<Policy Name>”. The standard policy names are “Readers”, “Writers“, “Admins” and “Endorsers”. For example, “/Channel/Application/Org1/Readers” governs who can read the channel. Figure 5 shows our policies for Org1 (Org2 and IoT also have the same policies). The policy name is Readers, and the policy type is Signature. The rule for the Readers policy is ”OR“ which means that anyone from Org1MSP.admin, Org1MSP.peer, or Org1MSP.client can read the channel. Figure 6 shows our Orderer organization policies.

### 4.3. Resources Access Control Lists (ACLs)

**Figure 7:**
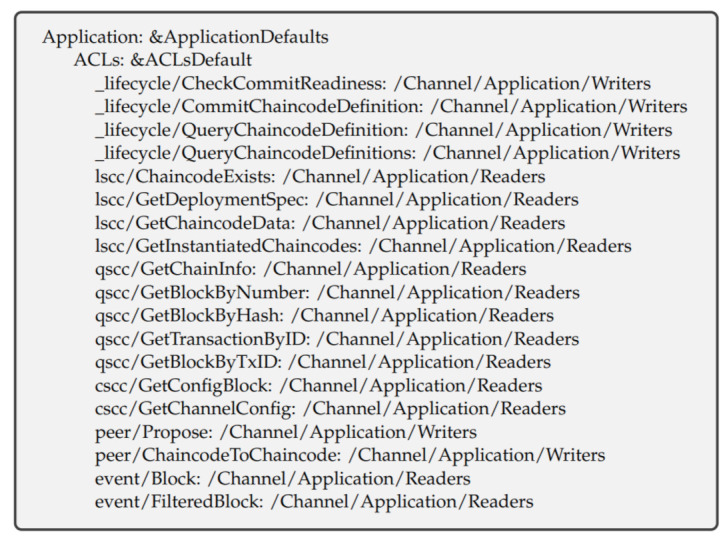
ACL policies declaration in the experimental network.

**Figure 8:**
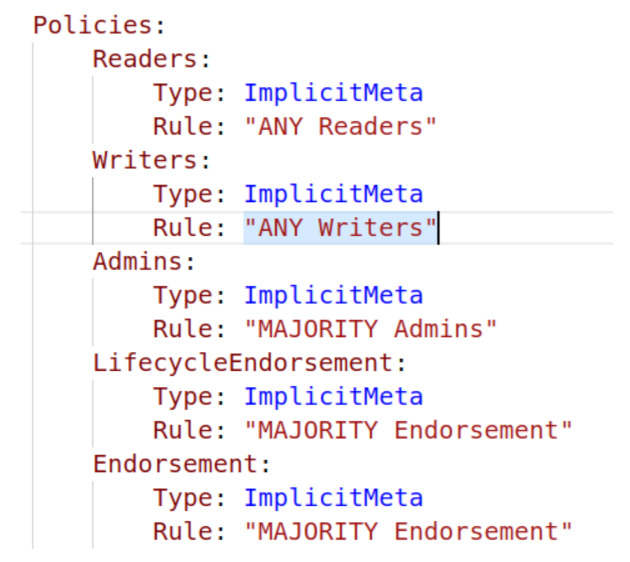
ACLs Implicit Meta policies for experimental network.

The access control lists allow managing access to resources with the definition of policies. The resources in our network are events sources and the functions exposed by the system chaincode and user chaincode. The ACLs can be created using signature or Implicit Meta policies. Figure 7 and Figure 8 show our ACLs policies for our experimental network. Policies are used to control who has access to which network resources. As seen in Figure 8, the fourth row from the bottom.


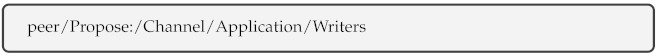


**Figure 9:**
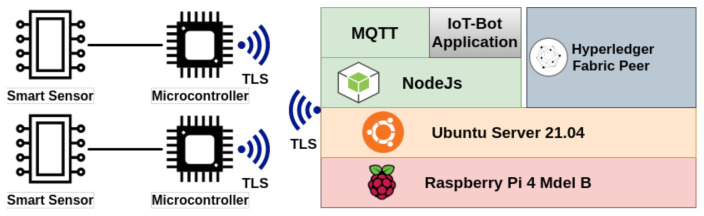
IoT-blockchain application collecting data from environment.

Represents the client’s chaincode resource. The writers policy for this resource is depicted in Figure 9. As shown in the mentioned figure, Implicit Meta is the policy for writers, and ”Any Writers“ is the rule. It means that any member organization’s writer can invoke the chaincode on the network. The organization’s policies regarding writers are described in Figure 6. The Org1 policy for writers defines a writer as any administrator or client of Org1. It implies that any user or client with the admin or client role can invoke chaincode. Assume that we deleted the policy rule “Org1MSP.client" from the writers policy. In this instance, only administrators may invoke the chaincode, and the client loses the writers privilege.

Fabric Policies enable us to specify access to our resources in accordance with the organization’s requirements. For instance, our Internet of Things (IoT) gadgets are sensitive. The consortium determined that only the administrator of the IoT organization has the authority to invoke the chaincode. We may specify this decision in our policy section as StrictAdminPolicy, using the policy type “Signature” and the rule “IoTMSP.admin”. We may point the writers’ resource to the StrictAdminPolicy.


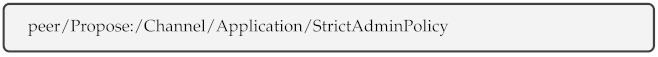


Now, only users or clients with the admin role in an IoT organization may invoke the chaincode on the network. We may adjust and establish policies to meet our organization’s security and operational requirements. To fine-tune access control, we must specify the logic for access control within our chaincode. For instance, only the admin has the ability to activate the chaincode. However, we want the chaincode to be invoked by a certain administrator with a specified id and attribute from a specific department. Such access control logic must be defined within the chaincode itself, as demonstrated in the coming stages.

### 4.4. Endorsement Policy

Users can set policies for chaincode execution in Hyperledger Fabric. These endorsement policies describe which peers must approve a transaction before it can be recorded. An endorsement policy is specified in a domain-specific language. Examples of endorsement policies are as follows:


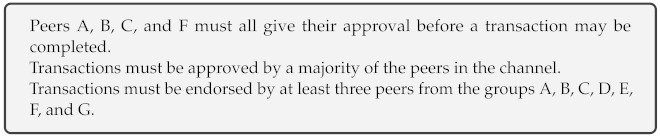


The default endorsement policy is is Implicit Meta with “Majority Endorsement" as shown in in Figure 7. According to our project requirements, the endorsement policy can be changed to type “Signature” and “IoTMSP.peer” only. We can override the endorsement policy on per chaincode basis while approving and committing the chaincode.

## 5. Implementation

We have implemented a use case from a real-world application. The IoT sensors are used to capture some environment data from the grain silo as shown in self describing Figure 9.

### 5.1. Architecture and Design

The experimental network to demonstrate the IoT-blockchain integration consists of four organizations, Org1, Org2, IoT and the Orderer organization. The application client accesses the temperature, humidity and the amount of NH3 from the MQTT broker and updates it to the blockchain network. To protect the network connection, the organizations’ TLS-CA server offers TLS (Transport Layer Security) to all blockchain components of the blockchain network, including the CA (Certification Authority) server and users. The CA server of the organization distributes X509 certificates to all components and actors in the organization’s blockchain network. The Hyperledger Fabric Certification authority and organization nodes for Org1, Org2, IoT and Orderer are hosted virtual machines on top of Xen Hypervisor with Xeon E5-2678-V3 X2 and 128 GB of RAM. Each hosted machine has x2 processing cores and 2 GB of RAM. The specifications of the nodes are given in Table 2.

**Figure 10:**
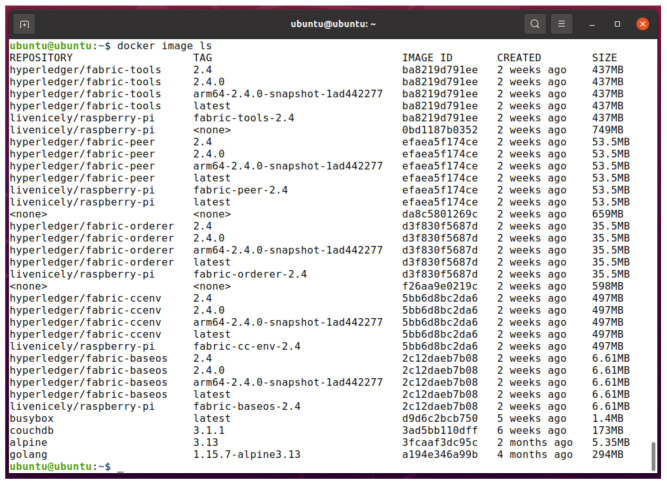
Hyperledger Fabric docker images on Raspberry Pi.

We used Raspberry pi 4 Model B with 8GB RAM as our IoT terminal with specifications in Table 3. The IoT terminal is based on 64 bit ARM architecture. We are using Ubuntu Server 21.04 as Operating System. The Hyperledger fabric images are not available for arm-based architecture. We have downloaded the source code and compile it to run Hyperledger Fabric on our IoT terminal with specifications as shown in Figure 10.

### 5.2. Deploy Chaincode

We installed our Silo Monitoring chaincode on the IoT terminal and other peer nodes. The chaincode is available online at GitHub [41]. The IoT terminal is based on ARM architecture, so we need to package the ARM architecture’s chaincode on the IoT device itself.

#### 5.2.1. Packaging and Installing Chaincode

The chaincode package phase involves creating a package (tar file) containing the chaincode and associated metadata. The package is appropriately labeled. The package may be done independently by organizations; it is more typical for one organization to develop and distribute it to all companies to verify that they all have the same chaincode.

The installation phase entails installing this package file on the peers. Only the peers that are engaged in chaincode invoke and query require installation of chaincode.

**Figure 11:**
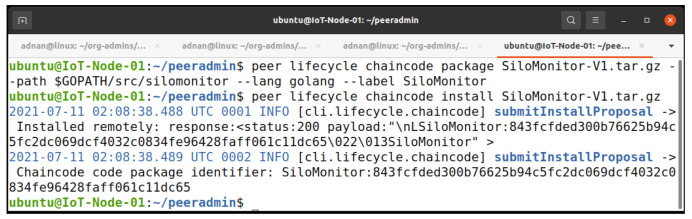
Package and install chaincode on Raspberry Pi IoT terminal.

Figure 11 illustrates the packaging and installation of our chaincode on the IoT terminal. It needs to package the chaincode on the IoT terminal itself due to the architecture difference. In second command, we have successfully installed the chaincode on the device. Similarly, we installed the chaincode on Org1 and Org2 peer nodes. Furthermore, the chaincode is unusable until it is not committed to the channel. The chaincode generates a package identification (Package ID) in the format <label>.hash> upon successful installation.

#### 5.2.2. Approve Chaincode Definition

**Figure 12:**
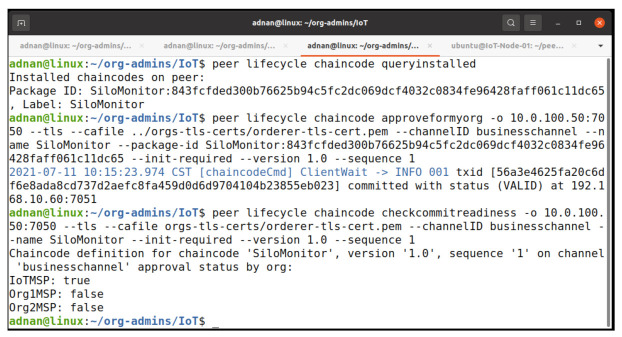
Approve chaincode and check commit readiness on peer node.

Organizations are needed to authorize the chaincode expressly by approving it for the organization. The lifetime endorsement policy governs the number of organizations required to approve the chaincode. The ordering service is engaged in the approval process since each approval generates a new block. This means that all peers are aware of the approval status. When approval is granted, we must indicate the channel to which the chaincode should be delivered. Certain information is necessary, such as a flag indicating if the chaincode contains executable Init() code. Once the requisite number of organizations has approved the chaincode, it is ready for commit. Figure 12 refers our procedure to approve the chaincode on the IoT terminal. In first step, we query the installed chaincode to obtain the package id, and then we approve the chaincode as org admin. In final step, we query to check if the chaincode is ready to commit on the network. The query results show that only IoTMSP accepted the chaincode on the network.

#### 5.2.3. Commit Chaincode on Channel

**Figure 13:**
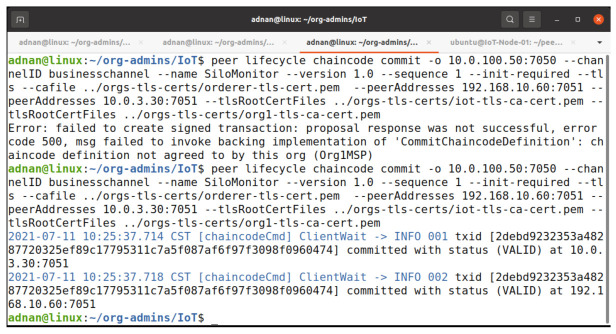
Commit chaincode transaction.

Any company can begin a chaincode commit. The organizations must first accept the chaincode. A new block is generated, and all peers commit it to the ledger. Figure 13 describes our step to commit the chaincode. We try to commit the chaincode, but it fails to meet the required endorsement policy, as the Org1 admin has not approved the chaincode on the network. In the next step, we commit it again after the approval of the Org1 admin. According to our endorsement policy, that is, “Majority Admins”, we are not required to include the Org2 peer to commit the chaincode but Org2 cannot participate in this chaincode until it does not approve and commits the chaincode for its organization.

#### 5.2.4. Endorsement Policy Testing

**Figure 14:**
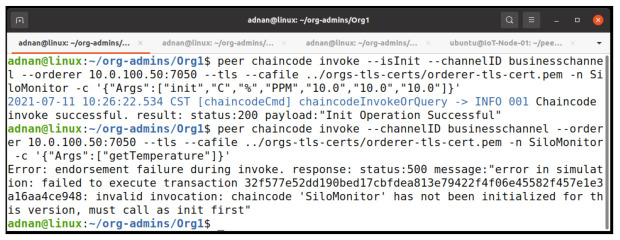
Signature policy failed on invoking the chaincode.

**Figure 15:**
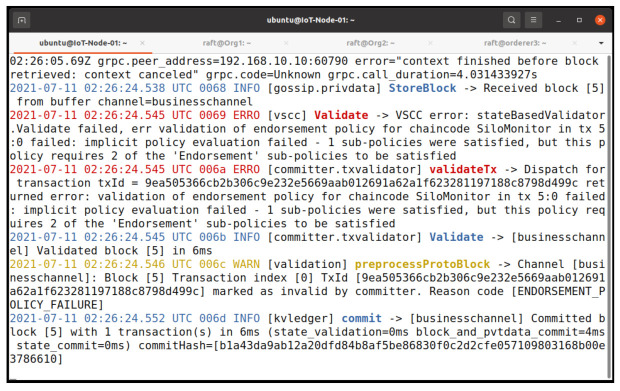
Signature policy failed log.

To further clarify our endorsement policy, which is the majority endorsement, we invoke our chaincode as the IoTMSP admin. Figure 14 shows the results of our invocation and query command. The invoke process is successful but it fails on obtaining the consensus, which results in the failure of the query because the invoke process is not committed on the network. Figure 15 shows the log of the peer. It shows that the endorsement fails during the policy evaluation.

**Figure 16:**
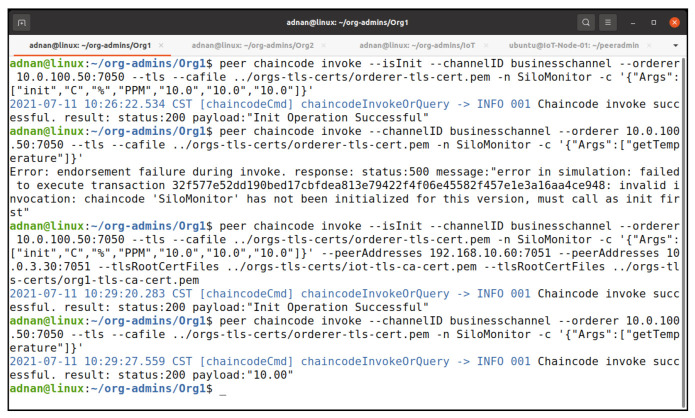
Chaincode invocation with majority endorsement peers.

**Figure 17:**
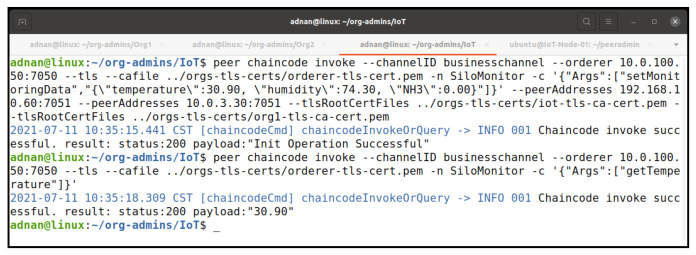
Successful chaincode invocation to update date as MSPIoT admin.

To meet the policy requirements, we selected IoTMSP peer and Org1MSP peer to invoke the chaincode again; this time, our invocation was successful (Figure 16). We can see the results of our query to obtain the temperature. We put another transaction as the IoTMSP admin to update our silo monitoring data, which resulted into the successful invocation of the chaincode and query (Figure 17).

### 5.3. Programmatic Access Control

#### 5.3.1. Client Identification Library

The Hyperledger Fabric policies are not enough to restrict access to the IoT device chaincode. We need to explicitly design the access control into the chaincode. The Hyperledger Fabric client identification library provides us the required functionality to program the access control into the chaincode. We explicitly need to import this library into our chaincode, as it is not the part of the core Hyperledger Fabric blockchain network functionality.

#### 5.3.2. Access Control Implementation

**Figure 18:**
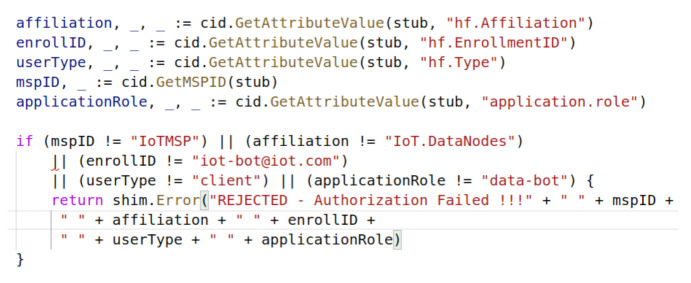
Chaincode version 2 programmatic access control.

**Figure 19:**
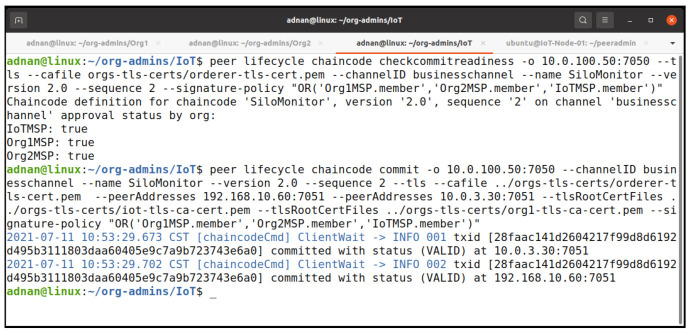
Committing chaincode version 2 on the network.

We updated our chaincode on version 2 and added some additional requirements using the client identification library functionality as shown in Figure 18. This time, we less restricted our endorsement policy and overrode the default policy during the approving and committing chaincode process. As a result, any member from any organization could endorse the chaincode for experimental purposes. Figure 19 shows the process of committing our chaincode v2 on the network. We did not need to initialized our chaincode again, as we have already initialized it during our first chaincode experiment.

### 5.4. Access Control Evaluation

**Figure 20:**
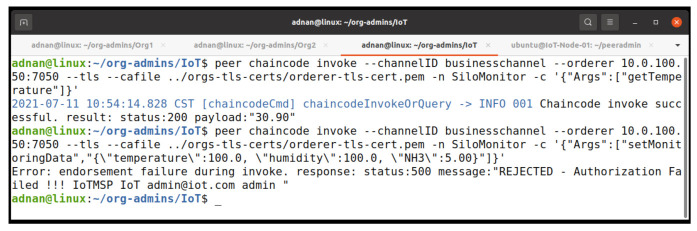
Authorization failed to invoke the chaincode.

We queried our chaincode to obtain the temperature and it was a success; however, we could not update our chaincode as the IoTMSP admin because it conflicted with our access control logic defined in our chaincode, shown in Figure 20. Only the IoT member with specific attributes could update the chaincode data. The certification authority is needed to certify an actor with the appropriate required permissions, as done in the next step.

#### Grant Attributes and Re-evaluation of Access Control

**Figure 21:**
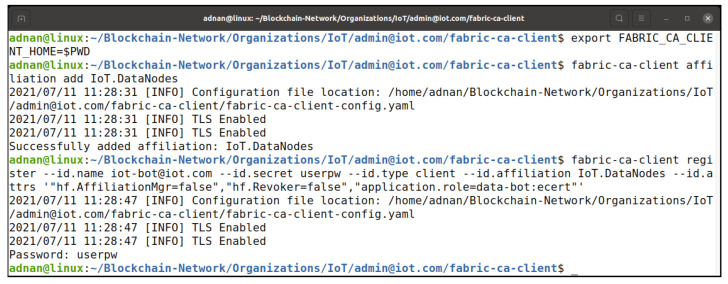
Fabric CA user registration process.

**Figure 22:**
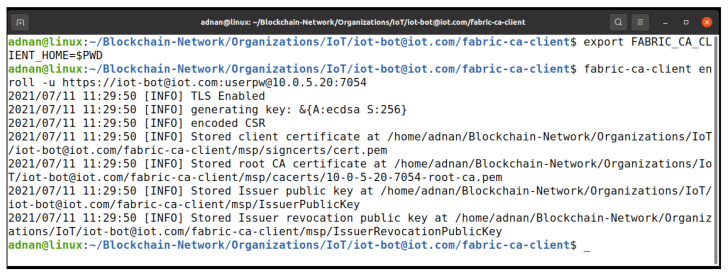
Fabric CA user enrollment process.

**Figure 23:**
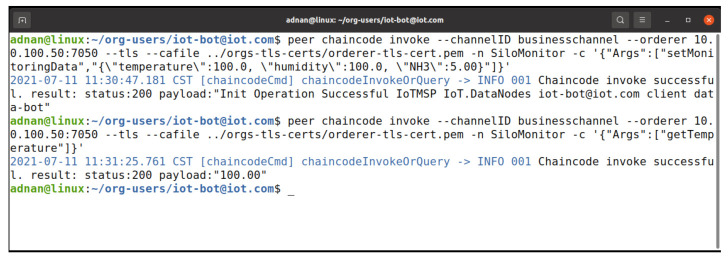
Access granted on the chaincode.

The IoTMSP organization admin needs to register the user with proper attributes to be able to access the chaincode update process. Figure 21 shows the process of registering the user with the required attributes, while Figure 22 shows the process of enrolling the user to obtain the X509 certificate to participate in the network. In Figure 23, the user iot-bot is granted access to update the chaincode data. The iot-bot id is able to update the data on the chaincode.

## 6. Performance Limitations

**Figure 24:**
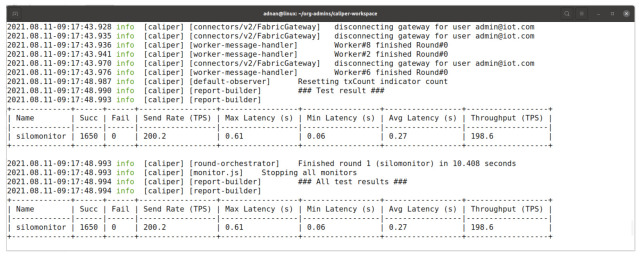
Hyperledger Caliper Benchmark Results.

In IoT devices like Raspberry Pi the overall performance of the device is depends on the memory we used, like the class of the memory card, USB storage or SSD storage in USB ports. A number of tools including Hyperledger Caliper is available for bench-marking. Currently we only measure the basic performance measurements using Hyperledger Caliper tool. We are getting about 200 TPS on reading temperature function. In multiple tests the maximum latency was 0.61 seconds while 0.06 seconds was the minimum latency and the average latency was 0.27 seconds Figure 24. The extensive and rigorous testing is plan in the future as describe in Future Studies section.

## 7. Conclusions

This paper discussed what solutions Hyperledger Fabric blockchain technology offers for IoT devices. With Hyperledger Fabric, we created a blockchain-based access control solution for IoT devices. The system utilizes Hyperledger Fabric to segregate people and devices using rules and programmatic access management built into the chaincode itself. We discussed the solution implementation and demonstrated its performance on a Raspberry Pi 4B node. We demonstrated the fabric policies, chaincode installation, chaincode invoking, and benchmarking of the solution. The study indicates that Hyperledger Fabric blockchain technology is pre-loaded with all the tools to manage IoT devices on the blockchain.

## 8. Future Work

Future work needs to experiments with an extensive network consisting of different IoT devices, including but not limited to different models of Raspberry Pi, Odroid XU4, Asus Tinker Board, and Nvidia Jetson nano. The system needs to evaluate different configurations and integration models to measure the performance matrices concerning hardware capabilities, processing and memory constraints, power consumption, different consensus protocols on the network, scalability, and network latency. It will define a model to decide where to deploy a full-scale server or edge server or use our already deployed IoT devices for various tasks, including consensus networks on the same devices.

## Figures and Tables

**Table 1:** Classification of Blockchains [15].

	Public Blockchain	Private Blockchain	Permissioned Blockchain
**Read Access**	No permission required from any authority	Read Access is private within organization participants	Public/Participants are permissible under some legal contract
**Write Access**	No permission required from any authority	Write Access is private within organization participants	Participants are permissible under some legal contract
**Consensus Process**	Anyone can join consensus process	Pre-selected nodes within organization	Pre-selected nodes within consortium

**Table 2:** Hyperledger Fabric nodes specifications.

Host	IP Address	OS	RAM	Fabric Version
IoT TLS-CA	10.0.5.10	Ubuntu Server 20.04 AMD 64	2 GB	Version: 2.3.1
IoT CA	10.0.5.20	Ubuntu Server 20.04 AMD 64	2 GB	Version: 2.3.1
peer@IoT	192.168.10.60	Ubuntu Server 21.04 ARM 64	8 GB	Version: 2.4.0
Org1 TLS-CA	10.0.3.10	Ubuntu Server 20.04 AMD 64	2 GB	Version: 2.3.1
Org1 CA	10.0.3.20	Ubuntu Server 20.04 AMD 64	2 GB	Version: 2.3.1
peer@org1	10.0.3.30	Ubuntu Server 20.04 AMD 64	2 GB	Version: 2.3.1
Org2 TLS-CA	10.0.4.10	Ubuntu Server 20.04 AMD 64	2 GB	Version: 2.3.1
Org2 CA	10.0.4.20	Ubuntu Server 20.04 AMD 64	2 GB	Version: 2.3.1
peer@org2	10.0.3.30	Ubuntu Server 20.04 AMD 64	2 GB	Version: 2.3.1
Orderer TLS-CA	10.0.100.10	Ubuntu Server 20.04 AMD 64	2 GB	Version: 2.3.1
Orderer CA	10.0.100.20	Ubuntu Server 20.04 AMD 64	2 GB	Version: 2.3.1
solo@orderer	10.0.100.50	Ubuntu Server 20.04 AMD 64	2 GB	Version: 2.3.1

**Table 3:** IoT Terminal Specifications.

Feature	Specifications
Platform	Raspberry Pi 4 Model B
Processor	64-bit Quadcore Coretex-A72
RAM	8GB LPDDR 4
OS	Ubuntu Server 21.04
IP Address	192.168.10.60
golang	go1.16.5 linux/arm64
Hyperledger Fabric	Version: 2.4.0
docker	Version: 20.10.7

## Data Availability

Data sharing is not applicable for this article.
